# Future liver remnant hypertrophy and postoperative outcomes: a retrospective comparison between segmental and main right portal vein embolization

**DOI:** 10.1186/s42155-025-00537-y

**Published:** 2025-03-28

**Authors:** Elif Can, Aboelyazid Elkilany, Sophia Paparoditis, Bernhard Gebauer, Dominik Geisel, Felix Krenzien, Anne Pohrt, Wibke Uller, Michael Doppler, Sebastian Ebel, Holger Gößmann, Uli Fehrenbach

**Affiliations:** 1 Department of Diagnostic and Interventional Radiology, Medical Center – University of Freiburg, Faculty of Medicine, University of Freiburg, Freiburg, Germany; 2 Department of Diagnostic and Interventional Radiology, University Hospital Leipzig, Leipzig, Germany; 3 Department of Diagnostic and Interventional Radiology, Charité-Universitätsmedizin Berlin, Corporate Member of Freie Universität Berlin, Humboldt-Universität Zu Berlin, Berlin Institute of Health, Berlin, Germany; 4 Department of General, Visceral and Transplantation Surgery, Charité-Universitätsmedizin Berlin, Corporate Member of Freie Universität Berlin, Humboldt-Universität Zu Berlin, Berlin Institute of Health, Berlin, Germany; 5 Berlin Institute of Health (BIH), Anna-Louisa-Karsch-Straße 2, 10178 Berlin, Germany; 6 Institute of Biometry and Clinical Epidemiology, Charité-Universitätsmedizin Berlin, Corporate Member of Freie Universität Berlin, Humboldt-Universität Zu Berlin, Berlin Institute of Health, Berlin, Germany

**Keywords:** Portal Vein Embolization (PVE), Future Liver Remnant (FLR), Liver Hypertrophy, Right hemihepatectomy, CIRSE classification, Clavien-Dindo Classification

## Abstract

**Objective:**

To assess the efficacy of segmental right portal vein embolization (SRPVE) versus main right portal vein embolization (MRPVE) in preoperative preparation for major hepatectomy.

**Methods:**

This retrospective single-center study included 220 consecutive patients who underwent portal vein embolization (PVE) before (extended) right hemihepatectomy between January 2014 and June 2021. Seventy-one patients underwent selective segmental embolization (SRPVE) and 149 patients underwent MRPVE. Volumetric analysis was conducted before PVE and before surgery. Key endpoints included evaluation of future liver remnant (FLR) hypertrophy, intraoperative complexity, and postoperative complications, technical success, clinical success, complications (Clavien-Dindo and CIRSE classifications), as well as evaluation of different factors which may influence hypertrophy of the FLR.

**Results:**

Technical success rate was 100% in the SRPVE group and 99.3% in the MRPVE group (*p* = 0.15). Clinical success rate was comparable between both techniques, measuring 95.8% in the SRPVE group and 95.3% in the MRPVE group (*p* = 0.18). Absolute hypertrophy (FLRabh) of the FLR was comparable between both techniques, measuring 47.15% in the SRPVE group and 40.78% in the MRPVE group (*p* = 0.54). Complication rates did not differ significantly (*p* = 0.12). Partial thrombosis involving the left portal vein, main portal vein, or mesentericosplenic region was observed in 2.8% of the patients in the SRPVE group vs 3.4% in the MRPVE group (*p* = 0.95). CIRSE Class II-VI complications were slightly higher in the MRPVE group (10.7% vs 9.8%, *p* = 0.82). Postoperative complications with Clavien-Dindo class ≥ IIIa occurred in 10.1% % in the MRPVE group vs 9.9% the SRPVE group (*p* = 0.92). Liver cirrhosis had a significant negative correlation with sFLR % increase following PVE (*r* = -0.54; *p* = 0.027). Neoadjuvant chemotherapy was also associated with reduced FLR hypertrophy following PVE, with a median sFLR% change of 63.8% (IQR: 60.8% – 75.2%) in patients who received neoadjuvant chemotherapy (*n* = 66 patients, 30%) compared to 82.6% (IQR: 77.4% – 84.2%) in those without chemotherapy (*n* = 154 patients, 70%).

**Conclusion:**

Selective segmental right portal vein embolization, sparing the main right portal vein, offers a safe and effective alternative to MRPVE, achieving comparable FLR hypertrophy while potentially simplifying intraoperative procedures and reducing postprocedural complications. Future research should focus on conducting large, prospective, multicenter trials to further compare the long-term outcomes of this technique, particularly with regard to liver regeneration, postoperative liver function, complications and overall survival.

## Introduction

Portal vein embolization (PVE) is a well-established preoperative technique to induce hypertrophy of the future liver remnant (FLR) and reduce the risk of post-hepatectomy liver failure (PHLF), particularly in patients requiring major liver resections [1–3]. This compensatory hypertrophy of the FLR is crucial in ensuring sufficient liver function after resection, particularly in patients with underlying liver disorders such as cirrhosis, cholestasis, or prior chemotherapy, which can impair liver regenerative capacity [3, 4].

The success of PVE in stimulating liver regeneration has been well-documented, with studies reporting FLR hypertrophy ranging from 30 to 50%, depending on patient-specific factors and technical approaches [5]. The magnitude of hypertrophy of the FLR depends on several factors, including the embolization technique, baseline liver condition, neoadjuvant chemotherapy, and the volume of liver resected [6–8]. However, the optimal technique for inducing FLR hypertrophy while minimizing complications remains a subject of ongoing investigation [7].

This study aims to evaluate and compare the outcomes of segmental right portal vein embolization (SRPVE), sparing the main right portal vein, with main right portal vein embolization (MRPVE) in patients undergoing (extended) right hemihepatectomy. The comparison focuses on future liver remnant (FLR) hypertrophy, intraoperative complexity, as well as postinterventional and postoperative complications. Specifically, this investigation explores whether sparing the main right PV achieves equivalent FLR hypertrophy while mitigating risks such as portal vein thrombosis. Additionally, it investigates whether this approach reduces overall procedural complexity and contributes to enhanced clinical outcomes.

## Patients and methods

### Patient population and study design

This retrospective, non-randomized, single-center study was conducted at a tertiary referral center for hepatobiliary surgery, from January 2014 to June 2021. The study included a total of 220 consecutive patients who underwent PVE prior to right or extended right hemihepatectomy. All patients were presented and discussed in an interdisciplinary tumor board (including a liver surgeon, hepatologist, radiologist, oncologist, and pathologist) where the indication for PVE followed by (extended) right hemihepatectomy was approved. The study was approved by the institutional review board. Informed consent was waived due to the retrospective nature of the study.

Inclusion criteria for PVE were based on future liver remnant thresholds of less than 20% in healthy liver, 30% in patients with chemotherapy-induced liver damage, and 40% in cirrhotic liver [2, 3]. Exclusion criteria included pre-existing macrovascular invasion, portal vein thrombosis, incomplete imaging follow-up, and contraindications for PVE such as severe liver dysfunction or systemic infection [4].

The cohort was divided into two groups:

- **MRPVE Group** (*n* = 149): Patients who underwent main right portal vein embolization, where both the distal right portal vein trunk and its segmental branches were embolized with coils.
- **SRPVE Group** (*n* = 71): Patients who underwent selective segmental right portal vein embolization with embolization of its segmental branches sparing the main right portal vein.

### Laboratory parameters and clinical scoring systems

Laboratory parameters performed within two weeks before PVE were systematically collected and analyzed including liver function tests (LFTs), coagulation profile. In addition, the Model for End-Stage Liver Disease (MELD) score was calculated for all patients before PVE [2, 9]. Furthermore, the results of LiMAx (Liver Maximum Capacity) test performed before and 4 weeks following PVE were collected and analyzed, with normal values ranging from 315–550 μg/kg/h. Patients with lower preoperative LiMAx values (< 150 μg/kg/h) were considered to be at high risk for postoperative liver dysfunction, and these patients were carefully monitored during the hypertrophic response following PVE [10, 11].

### Evaluation of different factors potentially influencing hypertrophy of the FLR

In addition, retrospective evaluation of different liver- and patient-related factors influencing hypertrophy of the FLR was conducted, including demographics, underlying tumor entity, presence of liver cirrhosis or cholestasis, biliary drainage, and neoadjuvant chemotherapy as well as laboratory values (LFTs) and liver function tests (MELD score and LiMAX test).

### Technique of portal vein embolization

Right PVE was performed using an ipsilateral percutaneous transhepatic access via ultrasound-guided puncture of one of the segmental branches of the right PV (RPV) using Neff Percutaneous Access Set (21-gauge, 15 cm length, Cook Medical, Bloomington, IN, USA) or 21-gauge Chiba needle (15 cm length). A 0.018" guide wire was advanced through the needle into the superior mesenteric vein or the splenic vein. Subsequently a4F introducer sheath (23-cm length, opaque tip; Britetip sheath; Cordis, Bridgewater, NJ, USA) was inserted via Seldinger technique to definitively establish access to the PV for the further angiographic intervention. Portography was performed to visualize portal vein anatomy. A reverse catheter (Sidewinder 1; Cordis) provided a retrograde access to the RPV [12].

**Fig. 1:**
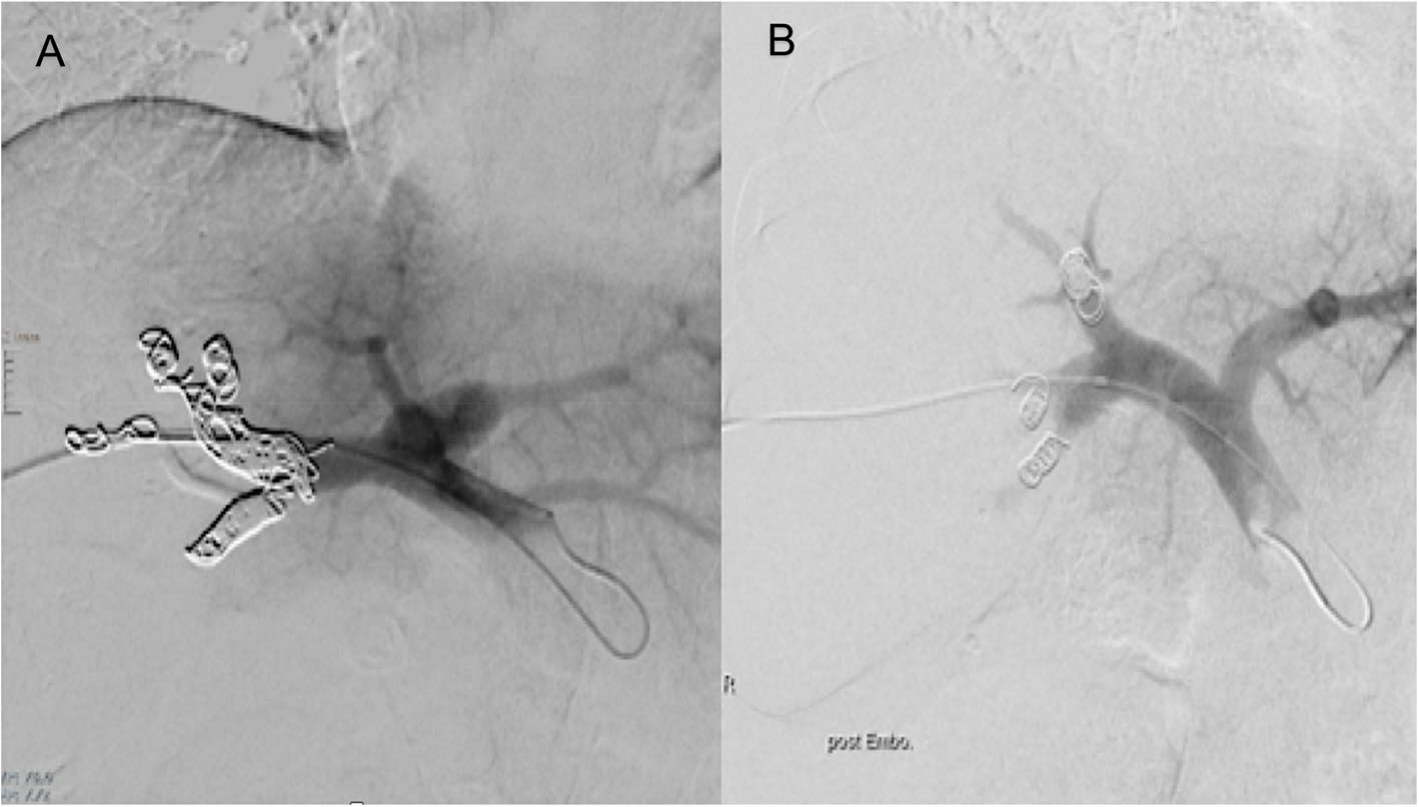
Main Right and Segmental and Portal Vein Embolization. **A** patient with perihilar cholangiocarcinoma who underwent embolization of the segmental right portal vein branches, as well as the right main portal vein (MRPVE group) using PVA particles and coils. **B** patient with perihilar cholangiocarcinoma with selective embolization performed at the origin of the segmental right portal vein branches, using PVA particles and coils (SRPVE group)

In the group of patients in whom only the segmental right PV branches were embolized (SRPVE group, *n* = 71 patients), embolization was performed selectively in the segmental branches of the right PV using Polyvinyl Alcohol (PVA) particles [500—710-μm particles (Contour, Boston Scientific, Natick, MA, USA) until stasis was reached] followed by selective coil embolization proximal in the segmental right PV branches using various 0.035" coils (Tornado, Cook Medical, and Interlock, Boston Scientific) sparing the main trunk of the right PV (Fig. 1) until stasis was achieved [12].

In the group of patients in whom PVE included embolization of the main trunk of the right PV (MRPVE group, n = 149 patients), additional large coils (Tornado or Nester (Cook Medical, Bloomington, IN, USA) as well as Interlock, Boston Scientific) or/and Amplatzer vascular plug type I or II (10–18 mm, St. Jude Medical, St. Paul, MN, USA, oversized by at least 20%), were used to embolize the distal main right PV if a distance from the coils/plug to the exiting main PV trunk of the FLR of at least 1 cm could be maintained (Fig. 1).

PVE was performed embolizing segments V–VIII and sparing segment I–IV (Branches to liver segment IV were not embolized unless explicitly requested by the treating surgeons). In the present study no patients with additional embolization of left-sided segment IV portal vein branch were included.

Portography was performed to document the final result of the embolization. During retraction of the sheath, the puncture channel was sealed with 2 mL of fibrin glue (Tisseel Fibrin Sealant or Tissucol Duo; Baxter, Deerfield, IL, USA) to avoid puncture-site bleeding or bile leakage [12].

### Volumetric analysis and hypertrophy outcomes

Dynamic contrast-enhanced computer tomography (DCE-CT) or magnetic resonance imaging (DCE-MRI) of the liver performed no more than two weeks prior to PVE and four to six weeks following PVE were analyzed to verify the indication of PVE and to assess hypertrophy and possible tumor progression before hepatectomy. A semi-automated volumetric analysis was performed by the same experienced reader (7 years gastrointestinal/abdominal imaging) using a commercially available software (Visage software, Version, 7.1.11 Visage Imaging, Berlin, Germany). CT-based volumetric analysis was conducted in the majority of patients (198/220) while MRI-based volumetric analysis was performed in only 22 patients in whom preoperative imaging was exclusively conducted using MRI. Liver volumes were estimated by manual delineation of the liver borders slice by slice in the 5 mm thick slices in CT or T1-VIBE sequence acquired approximately 20 min after gadoxetic acid administration (in the hepatobiliary phase of gadoxetic acid enhanced MRI), excluding large vessels and tumor masses [13]. For larger intrahepatic tumor masses or liver cysts, their volumes were measured separately and then subtracted from the total volume [13].

The following volumetric values were evaluated and analyzed:

- Total estimated liver volume (TELV) before and after PVE [14]
- Future liver remnant volume (FLRV) before PVE = Volume segment II + III before PVE
- Future liver remnant volume (FLRV) after PVE = Volume segment II + III after PVE
- Standardized FLR (sFLR) before PVE (%) = $\frac{FLR before PVE}{TELV before PVE}$ × 100 [14]
- Standardized FLR (sFLR) after PVE (%) = $\frac{FLR after PVE}{TELV after PVE}$ × 100 [14].
- FLR absolute hypertrophy [FLRabh (%)]: [13, 15]

$$\text{FLRabh} \% = (\frac{\text{FLRV after PVE }-\text{ FLRV before PVE }}{FLRV before PVE}) \times 100$$

- sFLR before PVE / sFLR after PVE (%) [15]

### Study definitions and endpoints

Primary endpoints were evaluation of hypertrophy outcomes of the FLR following PVE in both groups, as well as evaluation of technical success and clinical success of PVE. Secondary endpoints were evaluation of postinterventional and postoperative complications in both groups as well as evaluation of different factors which may influence hypertrophy of the FLR.

Technical success was defined as complete embolization of the targeted right portal vein branches without non-target embolization [15]. Clinical success after PVE was defined as achievement of sufficient volume and ratio of the FLR for the intended hepatectomy [15, 16]. Complications following PVE and posthepatectomy were classified using the CIRSE and Clavien-Dindo classification systems, respectively [17, 18].

### Statistical analysis

Statistical analysis was performed using IBM SPSS Statistics 27 (IBM Corp., Armonk, NY, USA). Continuous variables were expressed as means ± standard deviations (SD) or medians with interquartile ranges (IQRs), depending on data distribution. Independent sample t-test was used to compare continuous variables and chi-square tests to compare categorical variables. Chi-square test was to compare the two groups across the three major post-interventional complications and the CIRSE classification grade. Univariate and multivariate regression analyses were performed including different liver- and patient-related parameters to identify independent predictors of FLR hypertrophy. A p-value < 0.05 was considered statistically significant.

## Results

### Patient demographics and clinical characteristics

This retrospective study included 220 consecutive patients who underwent preoperative PVE, comprising 71 patients who underwent Segmental Right Portal Vein Embolization (SRPVE) and 149 patients who received Main Right Portal Vein Embolization (MRPVE). The gender distribution showed a higher percentage of males in the MRPVE group (62%) compared to the SRPVE group (51%), with no significant difference (*p* = 0.09). The mean age was similar across both groups, with 61.7 ± 11.4 years for SRPVE and 62.3 ± 11.7 years for MRPVE (*p* = 0.89). Neoadjuvant chemotherapy was administered to 19 (26.8%) patients in the SRPVE group and 47 (31.5%) in the MRPVE group (*p* = 0.87). The prevalence of liver cirrhosis was comparable between the two groups (35.2% in SRPVE vs. 38.9% in MRPVE; *p* = 0.72). Perihilar cholangiocarcinoma was the most common indication of (extended) right hemihepatctomy, present in 26 patients (36.6%) in the SRPVE group and 59 patients (39.6%) in the MRPVE group. There was no significant correlation between FLRV and gender (*P* = 0.70) or age (*P* = 0.09). Mean time interval between PVE and the preoperative imaging scan was 23.2 ± 11.4 days in the SRPVE group and 24.6 ± 13.1 days in the MRPVE group (*p* = 0.71). The demographic characteristics are summarized in Table 1.

**Table 1:** Patient Demographics and Clinical Characteristics

Variables	Segmental right PVE (*n* = 71)	Main right PVE (*n* = 149)	*p*-value
**Female / Male**	35 (49%) / 36 (51%)	56 (38%) / 93 (62%)	0.09
**Age (years); mean ± SD (range)**	61.7 ± 11.4 (34–84)	62.3 ± 11.7 (28–77)	0.89
**BMI (kg/m**^**2**^**); mean ± SD (range)**	25.7 ± 2.1 (20.2 – 30.8)	26.3 ± 2.3 (21.4 – 32.6)	0.23
**Neoadjuvant Chemotherapy**	19 (26.8%)	47 (31.5%)	0.87
**Liver cirrhosis**	25 (35.2%)	58 (38.9%)	0.72
**Cholestasis**	25 (35.2%)	58 (38.9%)	0.73
**Biliary drainage**	23 (32.4%)	47 (31.5%)	0.15
**LiMAX before PVE (μg/kg/h)**	330.46 ± 113.64	369.65 ± 122.62	0.09
**MELD Score**	11.75 ± 2.7	9.33 ± 3.1	0.82
**ALT (U/L)**	68.44 ± 10.50	57.22 ± 9.00	0.37
**AST (U/L)**	35.11 ± 4.50	41.47 ± 5.50	0.52
**AP (U/L)**	119.44 ± 22.7	158.30 ± 24.3	0.13
**GGT (U/L)**	115.33 ± 25.30	255.41 ± 32.6	0.08
**Tumor entities / Indication for (extended) right hemihepatectomy**			0.92
Perihilar cholangiocarcinoma	26 (36.6%)	59 (39.6%)	
iCCa	16 (22.5%)	28 (18.8%)	
CRC	21 (29.6%)	48 (32.2%)	
HCC	5 (7.0%)	9 (6.0%)	
Metastasis	2 (2.8%)	3 (2.0%)	
Other	1 (1.4%)	2 (1.3%)	

*BMI* Body Mass Index, *LiMAX test* maximum liver function capacity, *PVE* Portal Vein Embolization, *MELD score* Model for End-Stage Liver Disease Score, *ALT* Alanine Aminotransferase, *AST* Aspartate Aminotransferase, *AP* Alkaline Phosphatase, *GGT* Gamma-Glutamyl Transferase, *iCCa* Intrahepatic Cholangiocarcinoma, *CRC* Colorectal Cancer, *HCC* Hepatocellular Carcinoma

### Volumetric analysis and postoperative data

**Fig. 2:**
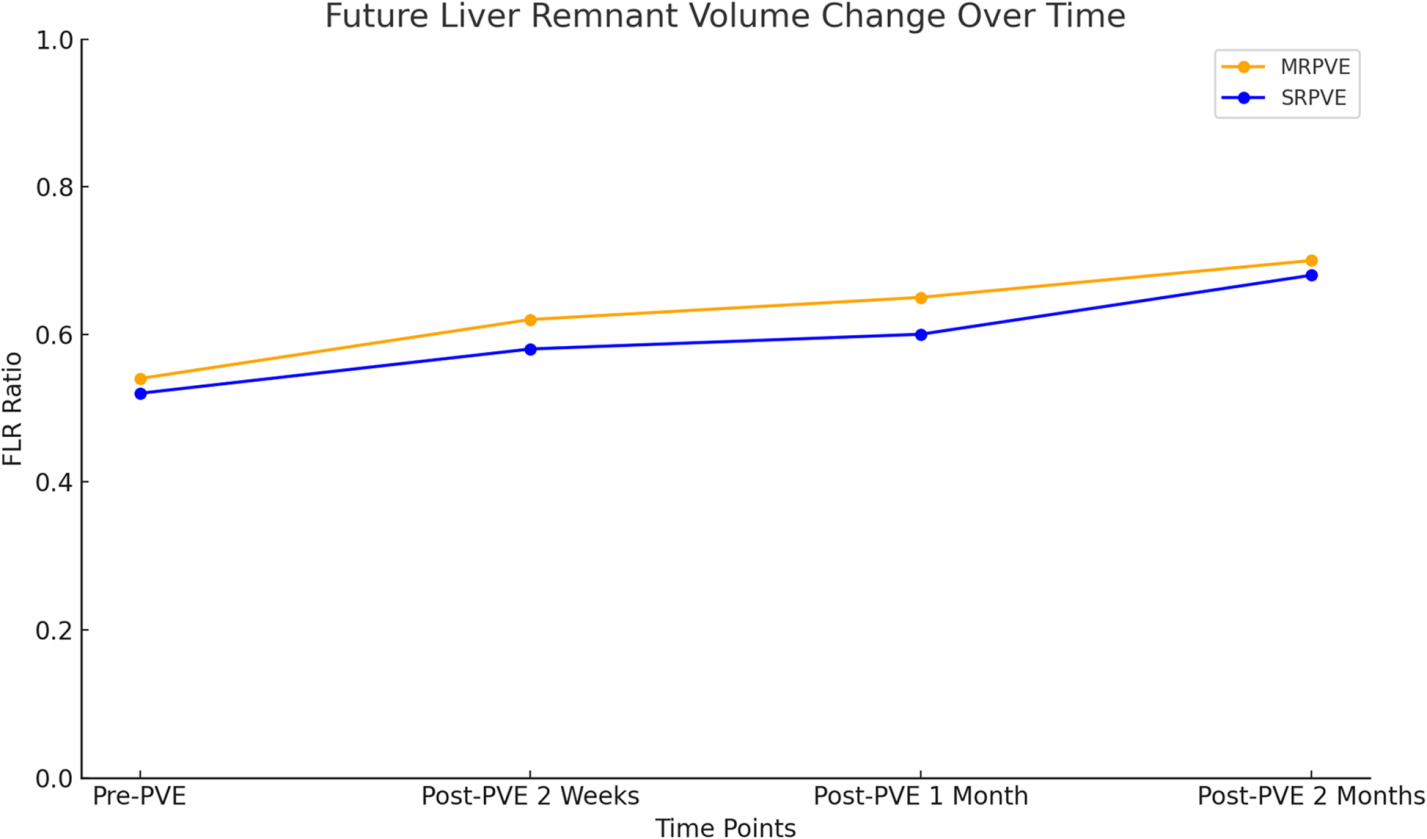
Change in standardized Future Liver Remnant Volume (sFLR) over time This graph illustrates the changes in the FLR ratios (sFLR before PVE / sFLR after PVE (%)) from pre-PVE to the measurements taken at two weeks, 1 and 2 months post-PVE. SRPVE; Segmental Right Portal Vein Embolization, MRPVE; Main Right Portal Vein Embolization

Volumetric analysis demonstrated comparable hypertrophic responses of the FLR between techniques. The FLRV before PVE was 271.13 ± 107.20 cm^3^ in the SRPVE group and 293.49 ± 136.25 cm^3^ in the MRPVE group (p = 0.19). After PVE, FLRV was 398.97 ± 127.83 cm^3^ for SRPVE and 412.19 ± 162.58 cm^3^ for MRPVE (*p* = 0.51). The standardized FLR (sFLR) before PVE was 17.13 ± 5.62% for the SRPVE group and 17.91 ± 6.88% for the MRPVE group (*p* = 0.37). After PVE, sFLR increased to 25.30 ± 7.68% in the SRPVE group and 25.23 ± 8.98% in the MRPVE group, with no significant difference between both groups (*p* = 0.95). Although FLRabh was numerically higher in the SRPVE group (47.15 ± 42.08% vs 40.78 ± 60.23% in the MRPVE group), the difference was not statistically significant (*p* = 0.54), suggesting comparable hypertrophic responses between techniques. The volumetric data are summarized in Table 2, Fig. 2.

**Table 2:** Volumetric analysis and postoperative data

	**Segmental right PVE (***n*** = 71)**		**Main right PVE** **(***n*** = 149)**		*p***-Value**
	Mean	SD	Mean	SD	
**Volumetric Analysis:**					
TLV before PVE (cm^3^)	1661.24	789.66	1692.99	809.64	0.78
TLV after PVE (cm^3^)	1634.97	524.95	1660.02	409.42	0.72
FLR before PVE (cm^3^)	271.13	107.20	293.49	136.25	0.19
FLR after PVE (cm^3^)	398.97	127.83	412.19	162.58	0.51
sFLR before PVE (%)	17.13	5.62	17.91	6.88	0.37
sFLR after PVE (%)	25.30	7,68	25,23	8.98	0.95
sFLR before PVE / sFLR after PVE (%)	69.02	16.07	72.05	16.05	0.19
FLRabh (%)	47.15	42.08	40.78	60.23	0.54
**Operative and postoprative data**					
Length of surgery (minutes)	180.7	142.37	222.19	155.67	0.59
Length of hospitalisation (days)	16.28	17.42	21.39	21.61	0.36
Length of ICU Stay (days)	7.2	2.4	10.4	3.7	0.42
Overall survival (months)	6.23	14.72	4.21	8.40	0.21

*PVE* portal vein thrombosis, *TLV* total liver volume, *FLR* future liver remnant (Segment II + III), *sFLR* standardized FLR, *FLRabh* FLR absolute hypertrophy

Technical success rate was 100% in the SRPVE group and 99.3% in the MRPVE group (*p* = 0.15). Clinical success rate was comparable between both techniques, measuring 95.8% in the SRPVE group and 95.3% in the MRPVE group (*p* = 0.18). The threshold of standardized FLR (sFLR) ratio was 20% (colorectal metastases), 30% (colorectal metastases with postchemotherapy liver injury) and 40% (hepatocellular carcinoma with underlying liver cirrhosis and Klatskin tumours) [15, 16].

### Postinterventional complications

Tumor progression following PVE was observed in 11 patients (15.5%) in the SRPVE group and 12 patients (8.1%) in the MRPVE group, (p = 0.07).

The overall rate of postinterventional complications was comparable between both techniques, with a *p*-value of 0.12. Partial thrombosis involving the left portal vein, main portal vein, or mesentericosplenic region was observed in 2 patients (2.8%) in the SRPVE group and 5 patients (3.4%) in the MRPVE group (*p* = 0.95). The rate of CIRSE Class II-VI complications was slightly higher in the MRPVE group (14 patients, 10.7%) compared to 7 patients (9.8%) in the SRPVE group, with *p*-value of 0.82 suggesting no significant differences in complication severity between the two techniques. Postinterventional complications are summarized in Table 3.

**Table 3:** Post-embolization und postoperative complications incuding CIRSE and Clavien-Dindo classifications

	**Segmental right PVE (n = 71)**	**Main right PVE** **(n = 149)**	**p-value**
**Tumor progression following PVE**	11 (15.5%)	12 (8.1%)	0.07
**Postinterventional Complications:**			0.12
Partial Thrombosis of the Main or Left PV	2 (2.8%)	5 (3.4%)	0.95
Subcapsular Biloma	4 (5.6%)	5 (3.4%)	0.11
Subcapsular Hematoma	4 (5.6%)	4 (2.7%)	0.73
Liver Abscess	1 (1.4%)	4 (2.7%)	0.08
Extrahepatic Displacement of Embolic Agent	0 (0%)	1 (0.7%)	0.15
**CIRSE Classification:**			0.82
I (%)	4 (5.6%)	5 (4.7%)	
II (%)	3 (4.2%)	6 (4.0%)	
III (%)	3 (4.2%)	5 (4.7%)	
IV (%)	1 (1.4%)	3 (2.0%)	
V (%)	0 (0%)	0 (0%)	
VI (%)	0 (0%)	0 (0%)	
**Postoperative complications:**			0.23
Bile leckage	7 (9.9%)	15 (10.1%)	0.57
Cholangitis	1 (1.4%)	3 (2.0%)	0.46
CBD Injury	0 (0%)	1 (0.7%)	0.67
Subcapsular Biloma	4 (5.6%)	11 (7.4%)	0.62
PHLF	5 (7.0%)	5 (3.4%)	0.22
Liver Infarct / Hepatic Perfusion Deficit	0 (0%)	5 (3.4%)	0.18
Postoperative Bleeding	4 (5.6%)	5 (3.4%)	0.43
Gastroparesis	2 (2.8%)	2 (1.3%)	0.45
Ileus	3 (4.2%)	7 (4.7%)	0.87
Sepsis	7 (9.9%)	10 (6.7%)	0.42
**Clavien-Dindo Classification:**			0.92
I (%)	1 (1.4%)	3 (2.0%)	
II (%)	3 (4.2%)	4 (2.7%)	
IIIa (%)	3 (4.2%)	9 (6.0%)	
IIIb (%)	1 (1.4%)	1 (0.7%)	
IVa (%)	1 (1.4%)	1 (0.7%)	
IVb (%)	0 (0%)	0 (0%)	
V (%)	2 (2.8%)	4 (2.7%)	

*PV* Portal Vein, *CIRSE* Cardiovascular and Interventional Radiological Society of Europe, *PHLF* Posthepatectomy livr failure, *CBD* Common bile duct

### Postoperative complications

The incidence of postoperative complications stratified according to the Clavien-Dindo classification was comparable between both techniques (*p* = 0.92). Bile leakage was the most frequent postoperative complication, occurred in 7 patients (9.9%) in the SRPVE group and 15 patients (10.1%) in the MRPVE group (*p* = 0.57) while postoperative arterial compromise leading to segmental ischemic insults in the liver was not observed in the SRPVE group while was noted in 5 patients (3.4%) in the MRPVE group (*p* = 0.18). Furthermore, postoperative bleeding occurred in 4 patients (5.6%) in the SRPVE group compared to 5 patients (3.4%) in the MRPVE group (*p* = 0.43). The incidence of gastroparesis and ileus were similar between groups, with *p*-values of 0.45 and 0.87, respectively. Postoperative complications are summarized in Table 3.

### Factors influencing hypertrophy of the FLR following PVE

Liver cirrhosis had a significant negative correlation with FLR % increase following PVE (*r* = −0.54; *p* = 0.027), indicating that cirrhosis adversely affects hypertrophy. Patients with liver cirrhosis (*n* = 83, 37.7%) showed a median sFLR% change (sFLR before PVE / sFLR after PVE) of 66.1%, (IQR: 61.3%—75.9%) compared to 81.4%, (IQR: 73.3.3%—85.9%) in patients without liver cirrhosis (*n* = 137). This wider range suggests that non-cirrhotic patients had a greater variability in their response to PVE. Correlation analysis of variables affecting FLR hypertrophy is summarized in Table 4.

**Table 4:** Pearson correlation analysis of different variables affecting hypertrophy of the FLR following PVE

	**FLRabh (%)**	
**Variable**	**r**	*P***-value**
Age	−0.92	0.09
Gender	0.61	0.70
BMI	0.89	N/A
Liver Cirrhosis	−0.54	0.027
Chemotherapy	−0.54	0.14
LiMAX	0.67	0.93
MELD Score	−0.16	N/A

*BMI* Body Mass Index, *LiMAX test* maximum liver function capacity, *PVE* Portal Vein Embolization, *MELD score* Model for End-Stage Liver Disease Score

**Fig. 3:**
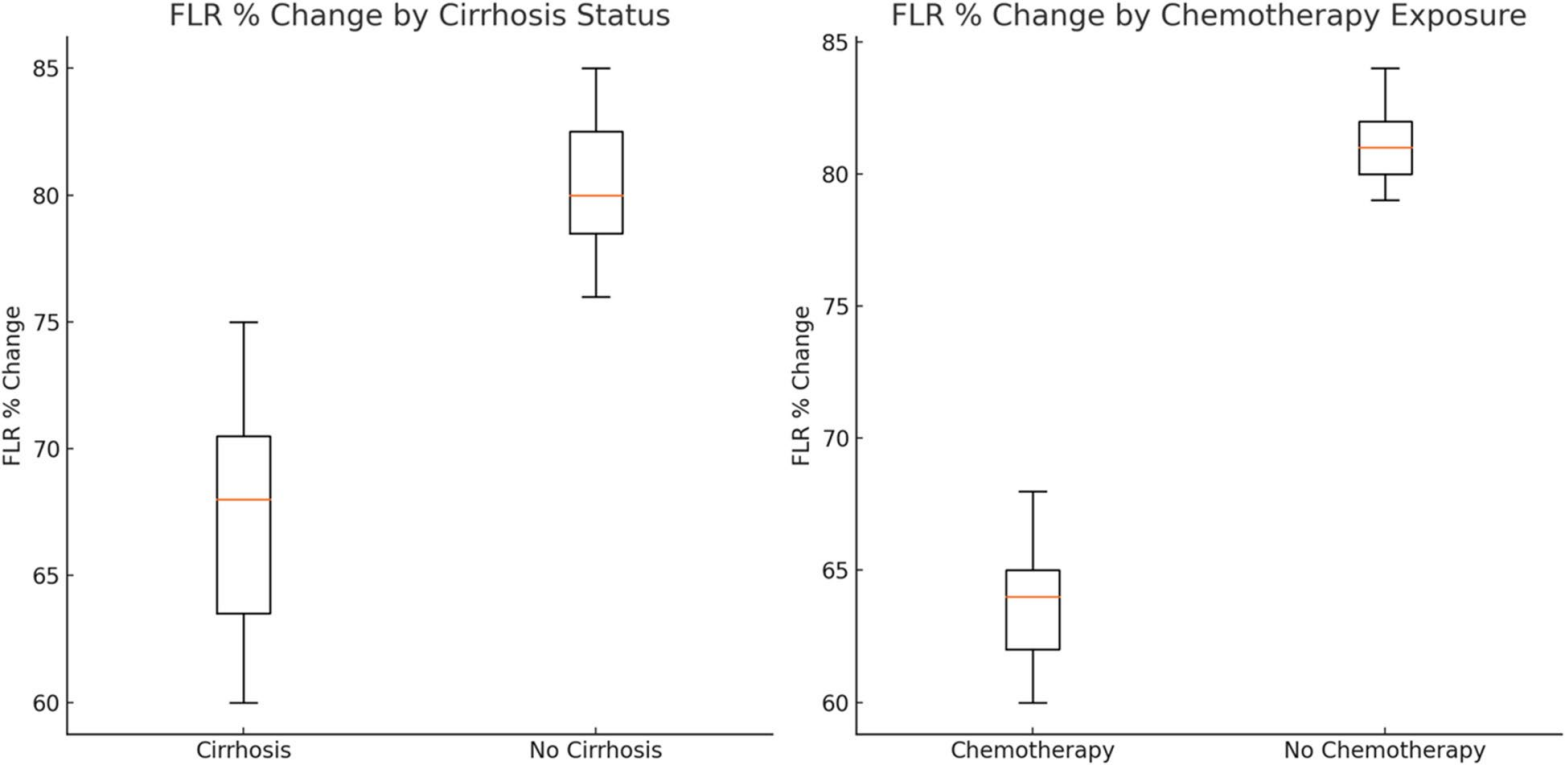
Box plots for sFLR % Change (sFLR before PVE / sFLR after PVE) by Patient Factors. **A** FLR % Change by Cirrhosis Status: This plot shows the variability in future liver remnant (FLR) percentage change between patients with cirrhosis and those without. **B** FLR % Change by Chemotherapy Exposure: This plot compares the FLR percentage change in patients who have received chemotherapy versus those who have not

Similarly, Patients who received neoadjuvant chemotherapy (*n* = 66 patients, 30%) showed a median sFLR% change of 63.8%, (IQR: 60.8%—75.2%) compared to 82.6%, (IQR: 77.4%—84.2%) in patients not exposed to chemotherapy (*n* = 154 patients, 70%). This suggests that chemotherapy negatively influence hypertrophy of the FLR following PVE with more favorable outcomes in terms of FLR hypertrophy observed in patients not exposed to neoadjuvant chemotherapy. Box plots for sFLR % Change is described in Fig. 3.

## Discussion

Even with the significant improvements in surgical techniques and better preoperative patient management as well as advancing postoperative critical care, major hepatectomy remains associated with a significant risk of morbidity and mortality, largely attributable to post-hepatectomy liver failure (PHLF), with reported incidences ranging from 1.2% to 32%. Risk factors for PHLF are closely linked to the FLR volume and the underlying liver status, including post-chemotherapy liver injury and chronic liver diseases such as cirrhosis and chronic cholestasis [13–15, 19].

Portal vein embolization is the standard of care for increasing the volume of the FRL and reducing the risk of PHLF. However, approximately 20% of patients who have received preoperative PVE do not advance to curative hepatectomy. This is primarily due to insufficient hypertrophy of the FRL, which poses an unacceptable high risk of PHLF, or because of tumor progression following PVE [13–15, 19].

This retrospective study aimed to evaluate the efficacy of selective segmental right portal vein embolization (SRPVE) versus embolization extending to the distal main right portal vein (MRPVE) in inducing hypertrophy of the FLR in patients undergoing preoperative preparation for major hepatectomy. The results showed that both techniques induced comparable hypertrophy of the FLR, but SRPVE was associated with fewer postinterventional complications, such as portal vein thrombosis, without compromising the hypertrophic response. To date, we could not identify any previous studies comparing these two techniques of PVE, which limits our ability to validate our findings.

In terms of procedural technique, SRPVE achieved comparable outcomes to MRPVE in both technical success or clinical success, with no significant differences between the two approaches. Technical success was not achieved in one patient in the MRPVE group due to displacement of the embolic agent in to left PV and splenic vein during PVE. However, the patient underwent right hepatectomy because the extent of thrombosis was not clinically significant. Clinical success was not achieved in a total of eight patients, including three patients with liver cirrhosis (one in the SRPVE group, two in the MRPVE group) and five patients with colorectal metastases and post-chemotherapy liver injury (two in the SRPVE group, three in the MRPVE group). Bilhim et al. reported comparable results with technical success rate of 98% and clinical success rate of 90–95% [1, 7, 15, 16, 20–26].

Selective embolization strategies could minimize postinterventional complications such as portal vein thrombosis and postoperative complications such as bile duct injury and bile leakage while achieving adequate hypertrophy of the FLR, aligning with our findings that SRPVE has lower adverse events [9]. On the other hand, it may be argued that hypertrophy of the FLR following additional embolization of the main right portal vein might be better due to diversion of a greater amount of portal flow, leading to more extensive hypertrophy of the FLR [2]. However, these results could be influenced by the underlying liver condition and the degree of fibrosis or cirrhosis.

In the present study, FLRabh was comparable between both techniques, measuring 47.15% in the SRPVE group and 40.78% in the MRPVE group (*p* = 0.54). Bilhim et al. demonstrated in the CIRSE Standards of Practice on PVE and double vein embolization/liver venous deprivation, after analyzing 9 articles investigating PVE, that the FLRabh ranged from 37% to 57 [15]. A systematic review conducted by Van Lienden et al. analyzed 44 articles involving 1,791 patients who underwent PVE and reported a mean FLRabh of 37.9 ± 0.1% (ranging from 20.5% to 69.4%) [20]. Similarly, a retrospective analysis by Kuo et al. observed a 35% increase in FLR volume in a retrospective review of 25 patients which is consistent with the present study [27].

Hypertrophy of the FLR is affected by several patient and liver related factors, especially the presence of chronic liver disease or neo-adjuvant chemotherapy [28–33].

Histopathological changes in the non-tumor liver parenchyma associated with chemotherapy could impair the regenerative capacity of the liver and consequently increase the risk of major liver resection.

Previous studies investigated liver hypertrophy after PVE in patients receiving chemotherapy. Goéré et al. stated that chemotherapy had no influence on hypertrophy of the FLR [30]. Beal et al. reported that patients who did not receive chemotherapy were more likely to have tumor progression between PVE and hepatectomy [31]. The authors concluded that although chemotherapy before surgery did not prevent liver hypertrophy after PVE, it did reduce it. However, a trend towards tumor progression was observed in patients who did not receive perioperative chemotherapy.

In the present study, tumor progression following PVE was observed in 11 patients (15.5%) in the SRPVE group and 12 patients (8.1%) in the MRPVE group, (*p* = 0.07). However, the degree of progression was not extensive to interfere with the decision of hepatectomy. Previous studies have indicated that PVE could potentially lead to tumor progression and poorer oncological outcomes in patients undergoing liver resection [32, 33]. The arterial buffer phenomenon, which involves increased arterial perfusion to the liver following portal vein occlusion, along with the release of hypertrophic factors involved in the regeneration process, may contribute to tumor growth [34]. However, larger systematic reviews and meta-analysis, revealed that there was no negative impact of PVE on local liver tumor progression or overall survival in patients who underwent liver surgery with prior PVE [35].

The insignificantly increased risk of partial thrombosis involving the left portal vein, main portal vein, or mesentericosplenic region observed in MRPVE might be attributed to the more extensive manipulation of the portal vein during the procedure, which could potentially increase the likelihood of vascular complications. These findings highlight the potential benefits of selective segmental PVE in reducing intraoperative procedural complexity and minimizing complications.

The overall rate of postinterventional complications was comparable between both groups. The rate of CIRSE Class II-VI complications was slightly higher in the MRPVE group, with p-value of 0.82 suggesting no significant differences in complication severity between the two techniques. A systematic analysis by van Lienden et al. [20] revealed that about 0.4% of the patients who underwent preoperative PVE did not proceed to the planned hepatectomy due to major complications such as portal or mesentericoportal venous thrombosis, liver abscesses, severe cholangitis, or sepsis.

There incidence of postoperative complications stratified according to the Clavien-Dindo classification was comparable between both groups (p = 0.92). Postoperative complications including postoperative bile leakage, arterial compromise leading to segmental ischemic insults of the liver, and postoperative bleeding were observed more in the MRPVE group, however, the difference between both techniques was not significant. The rate of postoperative complications was consistent with several previous studies investigating preoperative PVE with complication rates 5.8–21% as stated by Bilhim et al. [15].

### Limitations

As with any retrospective study, the present study is subjected to certain limitations. One key limitation is the potential for selection bias, as patients who underwent SRPVE versus MRPVE were not randomized. The choice was primarily based on preference of the interventional radiologist. Additionally, the small sample size, while adequate for initial comparisons, may have limited the statistical power to detect smaller differences between the two techniques.

## Conclusion

In conclusion, selective segmental right portal vein embolization, sparing the main right portal vein, offers a safe and effective alternative to MRPVE, achieving comparable FLR hypertrophy while potentially simplifying intraoperative procedures and reducing postprocedural complications. Future research should focus on conducting large, prospective, multicenter trials to further compare the long-term outcomes of this technique, particularly with regard to liver regeneration, postoperative liver function, complications and overall survival.

## Data Availability

The datasets used and/or analysed during the current study are available from the corresponding author on reasonable request.

## Abbreviations

SRPVE: Segmental right portal vein embolization
MRPVE: Main right portal vein embolization
PVE: Portal vein embolization
FLR: Future liver remnant
FLRabh: Absolute hypertrophy of the FLR
PHLF: Post-hepatectomy liver failure
LFTs: Liver function tests
MELD score: Model for End-Stage Liver Disease
LiMAx test: Liver Maximum Capacity test
PVA: Polyvinyl Alcohol
DCE-CT: Dynamic contrast-enhanced computer tomography
DCE-MRI: Dynamic contrast-enhanced magnetic resonance imaging
TELV: Total estimated liver volume
FLRV: Future liver remnant volume
sFLR: Standardized future liver remnant
IQRs: Interquartile ranges
